# Self-Harm and Suicidal Behaviors in Hong Kong Adolescents: Prevalence and Psychosocial Correlates

**DOI:** 10.1100/2012/932540

**Published:** 2012-04-01

**Authors:** Daniel T. L. Shek, Lu Yu

**Affiliations:** ^1^Department of Applied Social Sciences, The Hong Kong Polytechnic University, Hong Kong; ^2^Public Policy Research Institute, The Hong Kong Polytechnic University, Hong Kong; ^3^Department of Social Work, East China Normal University, Shanghai 200000, China; ^4^Kiang Wu Nursing College of Macau, Macau; ^5^Division of Adolescent Medicine, Department of Pediatrics, Kentucky Children's Hospital, University of Kentucky College of Medicine, Lexington, KY 40506, USA

## Abstract

The present paper examined the prevalence and psychosocial correlates of adolescent deliberate self-harm (DSH) and suicidal behavior in a representative sample of 3,328 secondary school students in Hong Kong. With reference to the previous year, 32.7% of the students reported at least one form of DSH, 13.7% of the respondents had suicide thoughts, 4.9% devised specific suicidal plans, and 4.7% had actually attempted suicide. Adolescent girls had significantly higher rates of DSH and suicidal behavior than did adolescent boys. Having remarried parents was related to an increased likelihood of DSH and suicide. While high levels of family functioning, overall positive youth development, and academic and school performance predicted low rates of DSH and suicidal behavior, cognitive and behavioral competencies were unexpectedly found to be positively associated with DSH and suicidal behavior. Theoretical and practical implications of the findings are discussed.

## 1. Introduction

Deliberate self-harm (DSH) and suicidal behaviors among adolescents represent major public health problems because they often lead to serious detrimental outcomes. Proximally, DSH and suicide bring physical harm (and even death) to the individual. Distally, DSH and suicidal behavior are related to a wide range of psychopathology, problem behaviors, and poor overall functioning. Although DSH and suicidal behaviors are closely associated, they are qualitatively different [1]. DSH is defined as the intentional injury of one's own body tissue without apparent suicidal intent while suicidal behavior refers to the act of deliberately or intentionally taking one's own life [2, 3]. Empirical studies conducted in the West have accumulated growing evidence on the rates and correlates of adolescent DSH and suicidal behaviors. In contrast, scientific information about the two phenomena among youth in different Chinese communities is sparse. Against the above background, the present study attempted to examine the prevalence and associated psychosocial factors of deliberate self-harm and suicidal behaviors in Chinese adolescents based on a large sample of secondary school students in Hong Kong.

In the past two decades, researchers have found that DSH has become more and more prevalent in adolescents, although the reported rates of DSH vary across countries. Using anonymous self-report questionnaires, Hawton et al. reported that 6-7% of the surveyed 15-year-old students in England showed at least one episode of DSH during the previous 12 months [4]. In the United States, lifetime prevalence of DSH generally ranged from 12% to 37.2% in secondary school students [5–7] and 12% to 20% in late adolescent and young adult populations [8, 9]. High rates of DSH have also been found in other Western countries. A prevalence rate of 24% was reported among young female adults in Italy [10] whereas a lifetime prevalence rate of 21.4% was found among 839 students in Turkey [11]. While DSH may occur at any age, research findings showed that adolescents and young adults are at a higher risk [12]. Thus, there is a consensus that adolescence is a risky period in which DSH may occur. 

As one of the leading causes of death in adolescents, suicide has always been a subject of continuing research across the world. Specifically, suicidal behavior can be viewed as existing along a continuum from thinking about ending one's life (suicide ideation or thoughts), developing a plan (suicide plan), nonfatal suicidal behavior (suicide attempt), to ending one's life (suicide) [13]. In recent years, although the rate of suicide directly resulting in adolescent death (fatal suicidal behaviors) decreases modestly, the prevalence of suicide ideation and suicide attempt (non-fatal suicidal behavior) has been found to be alarmingly high among youth. Based on a large sample of school students in Switzerland, researchers reported that about 26% of the 15- to 20-year-old adolescents had suicidal thoughts, 15% had suicidal plans, and 3% had suicide attempts [14]. In a systematic review of 128 studies, Evans, Hawton, Rodham, and Deeks revealed that on average 9.7% of adolescents reported that they had attempted suicide at some point in their lives and that an additional 29.9% of adolescents reported having suicidal thoughts at some point in their lives [15]. 

Although there are plenty of research-based data on the prevalence of DSH and suicidal behaviors in adolescents from Western countries, particularly the United States, such information is very limited from Asian countries including different Chinese communities. Existing research findings regarding the rates of DSH and suicidal behaviors in Chinese adolescents are equivocal because of the lack of systematic studies and numerous methodological variations, such as utilization of different assessment measures and procedures. For example, based on parent-reported questionnaires, Liu et al. found that 3.2% of a community sample of Chinese adolescents had deliberately harmed themselves [16] while a recent study using self-reported questionnaire showed that the prevalence rate of DSH was 15.5% among 2,013 Chinese students aged between 10 to 24 years [17].

 With reference to suicide, different prevalence findings were also reported. Liu investigated suicidal behaviors among 1,362 adolescents (mean age = 14.6 years) and found that 19.3% of the sample reported having suicidal ideation and 10.5% having suicide attempts in the past 6 months [18]. With a population-based sample, Pan et al. reported a similar rate of 18.6% of suicidal ideation in 23,976 students aged 12 to 19 years [19]. Yet, Tang et al.'s study showed apparently lower rates: suicidal ideation and suicidal attempts were reported by 8.8% and 3.5% of the participated Chinese adolescents [17]. In Hong Kong, 26.8% of a community sample of adolescents reported having ever thought about suicide in their life and 4.5% had suicidal ideation in the past 12 months; among the participants, 2.3% had made a suicide plan in the past year and nearly all of them actually attempted suicide [20]. Clearly, the existing mixed findings cannot provide an accurate picture of the situation of DSH and suicidal behavior in Chinese adolescents. As such, there is a great need for systematically examining the two problem behaviors with methodologically sound empirical research.

 To effectively prevent DSH and suicidal behaviors, numerous studies have been conducted to identify risk and protective factors for adolescents at different levels. At the individual level, gender was found to be a predictor of DSH. Adolescent girls had significantly higher self-harm rates than did adolescent boys [4, 21–23], and patients who utilized medical treatment for self-harm were more often females [24, 25]. Results regarding gender difference in suicide varied across studies and different suicide-related behaviors. In the United States, research findings have shown that males are four-times as likely to kill themselves and females are three- to nine-times as likely to have attempted suicide [26]. Several researchers suggest that adolescent males are prone to use more aggressive suicide methods which increase the likelihood of death [27]. In Hong Kong, Chan et al. reported that females had significantly higher rates of lifetime suicide ideation than did males [28]. However, it was also shown that there was no significant gender difference in suicide plans and suicide attempts in the past 12 months. 

At the family level, a number of studies suggest that different family factors are related to adolescent DSH and suicidal behaviors. First, most findings showed that low family economic status was associated with higher rates of both self-harm and suicidal behaviors among adolescents. For example, several researchers reported that low household income was significantly related to suicidal thoughts among Chinese adolescents [28, 29]. However, there are also reports suggesting no significant relationship between family financial problem and adolescent self-harm behaviors [30]. Second, there has been a link between parental marriage status and adolescent self-harm and suicide-related behaviors. In a longitudinal study, not living in a family with two biological parents at age 12 significantly predicted DSH three years later [31]. Researchers also found that divorce of parents can potentially trigger suicidal thoughts in young people [32]. Third, severe family dysfunctioning has been found to be a major factor in self-harming and suicide in adolescents. Lack of parent-adolescent communication, low levels of family cohesion and support, and family conflict were associated with adolescent suicidal ideation and DSH [33–35]. 

At the societal level, immigrant status has been considered a risk factor of adolescent suicide-related behaviors and DSH in different populations, mostly due to acculturation stress and insufficient support for immigrant youth. However, empirical findings are also inconclusive. Ponizovsky et al. compared suicide ideation and suicide attempts among immigrant Jewish adolescents from the former Soviet Union to Israel and two indigenous samples of Jewish adolescents in Russia and in Israel [36]. The results showed that the 6-month prevalence rate of suicidal ideation in the immigrant sample (10.9%) was significantly higher than that for Russian controls (3.5%) but not for Israeli natives (8.7%). On the other hand, an Australian study comparing native-born Australians, Australian-born children of immigrants, and immigrant adolescents did not find a higher risk of mental health problems among migrants [37]. With reference to Chinese population, although there are reports showing that immigrant youth in Hong Kong did not display more problem behaviors, such as Internet addiction, than did local adolescents [38], no study has directly examined whether immigrant status is associated with adolescent suicide-related behaviors and deliberate self-harm.

While the identification of risk factors for a problem behavior is critical for prevention purpose, it is equally important to identify protective factors that diminish the risk of developing the problem. Positive youth development has been increasingly recognized as a significant protective factor for youth risk behaviors. Researchers have documented evidence that different positive youth development constructs are negatively related to a wide range of problem behaviors in adolescents, including drug abuse, delinquency, and Internet addiction [38–41]. With reference to adolescent self-harm and suicidal behavior, several studies yielded associations of high self-esteem and high coping resources with low DSH. For example, McAuliffe et al. found that high self-esteem buffered the negative effect of passive-avoidant coping on self-harm [42]. Kwok Lai and Shek reported that suicidal ideation was positively related to hopelessness but negatively related to emotional competence and social problem solving in Hong Kong adolescents [43]. These findings suggest that youth suicidal behaviors and DSH may be prevented by strengthening positive youth development. Based on a comprehensive literature review, 15 positive youth development constructs have been identified [44], including bonding, resilience, social competence, recognition of positive behavior, emotional competence, cognitive competence, behavioral competence, moral competence, self-determination, self-efficacy, clear and positive identity, beliefs in the future, prosocial involvement, prosocial norms, and spirituality. However, there are no studies that directly examine the relationships between these constructs and adolescent DSH as well as suicide-related behavior. 

Although a large amount of work has been done to examine the phenomenon of adolescent DSH and suicidal behaviors, there are several problems intrinsic to the current studies. First, existing findings on DSH and suicidal behavior among adolescents are inconclusive. This may be due to the inconsistent measurement tools and various definitions of DSH and suicidal behaviors used in different studies. For example, while self-harm behaviors without suicidal intention should be differentiated from those with suicidal intention, there are studies in which the two different types of self-harm were mixed together. Second, most existing studies are cross-sectional and retrospective in nature, which leaves the causality of the relationship between different psychosocial factors and DSH as well as suicidal behaviors undecided. Third, small or unrepresentative samples were frequently used. Reliable prevalence data from large and representative samples would help researchers to gain a more accurate picture of adolescent DSH and suicide. Fourth, in comparison to the abundant information on adolescent suicide and DSH in Western countries, evidence of the prevalence and correlates of the DSH and suicidal behaviors in Chinese adolescents is far from enough. Fifth, empirical studies investigating the role of positive youth development in adolescent DSH and suicide are almost nonexistent. 

Against the above background, the present study attempted to examine the prevalence and psychosocial correlates of deliberate self-harm and suicidal behaviors among Chinese adolescents based on a large and representative sample of secondary school students in Hong Kong. This paper reports data collected at the first wave of a longitudinal study which aims at tracing youth development and determining the causal relationships between different psychosocial correlates and adolescent developmental outcomes. Deliberate self-harm was clearly differentiated from suicidal behaviors in terms of the existence of suicidal intention and a comprehensive measure which assesses various forms of self-harming behaviors was used. Suicidal behavior was operationally defined as behavior including suicide thoughts, suicide plan, and suicide attempt.

Specifically, this study focused on the following research questions. (1) What are the prevalence rates of DSH and suicidal behavior in Chinese adolescents in Hong Kong? (2) What are the relationships between gender, family economic status, family functioning, and Chinese adolescent DSH and suicide? Based on the literature, the following hypotheses were proposed: (a) females would have a higher level of DSH and suicidal behavior than males and (b) family economic status would be negatively related to adolescent DSH and suicidal behavior and (c) family functioning would be negatively related to adolescent DSH and suicidal behavior. (3) What is the relationship between positive youth development constructs and DSH as well as suicidal behavior among Chinese adolescents? It is expected that there would be negative relationship between positive youth development and adolescent DSH and suicidal behavior.

## 2. Methods

The present study is part of a large longitudinal study aiming at tracing the development and risk behaviors among Hong Kong adolescents over time. A total of 28 secondary schools in Hong Kong were randomly selected to participate in the study, with 5 schools from Hong Kong Island, 7 schools from Kowloon, and 16 schools from New Territories. Data regarding self-harming and suicidal behaviors collected in the first wave of this study are analyzed in this paper. 

### 2.1. Participants

All Secondary 1 students in the selected schools were invited to complete a questionnaire anonymously. There were 3,328 students responding to the questionnaire. The mean age of the participants was 12.59 years (SD = 0.74). These include 1,719 boys and 1,572 girls, with 37 students did not indicate their gender. While most students were born in Hong Kong (78.3%), there were 19.8% of the participants coming from mainland China and 1.9% from other places. Among the participated students, most had parents who both had a job (56.6%), 32.9% had either father or mother being employed, and 10.5% had parents who were both unemployed. The background demographic information of the participants is summarized in Table 1.

### 2.2. Procedures

In the school year of 2009-2010, the participants were invited to respond to a comprehensive youth development questionnaire including both existing instruments and scales developed by the first author. The questionnaire survey was conducted by a trained research assistant in classroom settings with standardized instructions. At each measurement occasion, the purposes of the study were introduced and confidentiality of the data collected was repeatedly ensured to all participants. School, parental, and student consent had been obtained before data collection. Participants responded to the questionnaires in a self-administered format. The research assistant was present throughout the administration process to answer possible questions from the participants.

### 2.3. Instruments

Participants were invited to respond to a composite questionnaire that comprises questions on demographic information, participants' family environment, family functioning, different measures of youth development constructs, and problem behaviors. For family environment, participants responded to questions regarding parental presence, educational level, parental marital status and family economic status. Family economic status is indexed by the question of whether the family of the participant is receiving comprehensive social security assistance (CSSA), financial aid provided by Hong Kong Government for low-income populations, at the time of survey, with 1 = received CSSA and 0 = did not receive CSSA. Students were also asked to indicate the marital status of their parents, including “divorced and not remarried,” “separate and not remarried,” “couple, first marriage,” “couple, second or above marriage,” and “others.” 

The scales used to assess family functioning, deliberate self-harming behaviors, suicidal behaviors, and positive youth development constructs are introduced below. Internal consistency of each measure for the present sample is summarized in Table 1. 

#### 2.3.1. Assessment of Family Functioning

Nine items were designed to provide information about three aspects of the participants' family functioning, including family mutuality, family conflicts, and family communication. Students were asked to respond to a five-point Likert scale on their perceptions of different aspects in their family lives. These items were abstracted from the Chinese Family Assessment Instrument [45]. Average score across the nine items was used as the indicator of general family functioning, with high scores representing for high levels of family functioning. Reliability analysis showed that the scale was internally consistent (Cronbach's *α* =.90).

#### 2.3.2. Deliberate Self-Harm Behavior Scale (DSHS)

This scale comprises 17 items that assess the occurrence of different deliberate self-harming behaviors of the participants in the past year, including cutting wrist, burning oneself, carving words or pictures into skin, scratching and biting oneself, rubbing sandpaper on body, dripping acid onto skin, using bleach or other chemical materials to scrub skin, sticking sharp objects and rubbing glass into skin, breaking bones, banging head against something to cause a bruise to appear, preventing wounds from healing, and other self-harm behaviors. Respondents answered yes (coded as 1) or no (coded as 0) to the 17 items according to their actual behavior in the past year. To differentiate self-harming behaviors without suicidal intention from suicide-related behaviors, a note that the behavior shall be conducted without suicidal intention is emphasized in each item. A composite score of DSHS was calculated for each individual by averaging the 17 item scores, which ranged from 0 to 1 with higher score representing more self-harm behaviors. Cronbach's alpha of the DSHS was 0.83 for the present sample.

#### 2.3.3. Suicidal Behavior Scale (SBS)

Participants' suicidal behaviors were measured by a four-item SBS in terms of three aspects: suicidal thought, suicidal plan, and suicidal attempt. The first item measured suicidal thought, which asked about whether the respondents had seriousy considered committing suicide in the past one year. The second item asked the participants whether they had made a specific plan for suicide, that is, suicidal plan. The third item enquired the number of actual suicide the participants had committed in the past year (0 = never; 1 = once to twice; 2 = three to four times; 3 = five to six times; 4 = seven to eight times; 5 = nine to ten times; 6 = more than ten times), that is, suicidal attempts. For the present study, this item was recoded: 0 = never attempted suicide; 1 = attempted suicide at least once. If the participants reported having attempted suicide, they were asked to indicate whether their suicidal behaviors need medical treatment in the fourth item. A composite score of SBS was computed by average scores of item 1, item 2, and the recoded item 3, which represents for a general suicidal tendency of the participants. In this study, Cronbach's alpha for the three items was 0.68. 

#### 2.3.4. Chinese Positive Youth Development Scale (CPYDS)

The CPYDS consists of 15 subscales which are listed as follows.

1. Bonding Subscale (three items)
2. Resilience Subscale (three items)
3. Social Competence Subscale (three items)
4. Emotional Competence Subscale (three items)
5. Cognitive Competence Subscale (three items)
6. Behavioral Competence Subscale (three items)
7. Moral Competence Subscale (three items)
8. Self-Determination Subscale (three items)
9. Self-Efficacy Subscale (two items)
10. Beliefs in the Future Subscale (three items)
11. Clear and Positive Identity Subscale (three items)
12. Spirituality Subscale (three items)
13. Prosocial Involvement Subscale (three items)
14. Prosocial Norms Subscale (three items)
15. Recognition for Positive Behavior Subscale (three items)

Based on factor analyses, Shek and Ma [46] proposed that the 15 subscales in the CPYDS could be further reduced to four dimensions.

- Cognitive behavioral competence (CBC): scale score was calculated by averaging scores on Cognitive Competence Subscale, Self-Determination Subscale, and Behavioral Competence Subscale.
- Prosocial attributes (PA): scale score was the mean score of Prosocial Involvement Subscale and Prosocial Norms Subscale.
- Positive identity (PIT): scale score was computed by averaging scores of Beliefs in the Future Subscale and Clear and Positive Identity Subscale.
- General positive youth development qualities (GPYDQ): scale score was the mean score of Resilience Subscale, Social Competence Subscale, Self-Efficacy Subscale, Moral Competence Subscale, Bonding Subscale, Recognition for Positive Behavior Subscale, Spirituality Subscale, and Emotional Competence Subscale.

Both the overall score of CPYDS, calculated by averaging all item scores in the scale, and the above four composite indicators were used to assess participants' positive youth development in the present study. Scores of the five indicators all range from 1 to 6 with higher scores representing high competences in the constructs. Descriptive statistics about all variables under study are listed in Table 1. It should be noted that although the administered questionnaire includes other subscales of the CPYDS, findings regarding the subscales will be reported elsewhere. The present paper only focused on the predictive effects of the overall CPYDS score and the four second-order positive youth development constructs on adolescent self-harming and suicidal behavior. Cronbach's alpha coefficients were  .96, .87, .83, .93, and  .87 for the whole CPYDS, CBC subscale, PA subscale, GPYDQ subscale, and PIT subscale, respectively.

#### 2.3.5. Academic and School Competence Scale

As a relatively independent positive youth development construct, participants' academic and school competence (ASC) were measured by three items. For the first item, participants were required to rate their perceived academic performance as compared to other peer students on a five-point Likert scale, with “1” = “very poor,” “2” = “below average,” “3” = “average,” “4” = “above average,” and “5” = “very good.” The second item asked the extent to which the respondents were satisfied with their academic performance (“1” = “very dissatisfied,” “2” = “dissatisfied,” “3” = “neutral,” “4” = “satisfied,” and “5” = “very satisfied”). The last question asked the participants to rate their conduct in school on a five-point Likert scale (“1” = “very poor,” “2” = “below average,” “3” = “average,” “4” = “above average,” and “5” = “very good”). The ASC scale score was calculated by averaging the item scores ranging from 1 to 5, with high scores representing for high academic and school competence. Reliability analysis showed that this measure was internally consistent (Cronbach's *α* =.67).

### 2.4. Data Analytic Plan

The first purpose of the present study was to provide a descriptive profile of different self-harm and suicidal behaviors among Hong Kong adolescents. Therefore, numbers and percentages of adolescents who reported different self-harming behaviors and suicidal attempts were first computed. Means and standard deviations of two quantified indicators, DSH and SB, were also summarized and compared by groups of gender, immigrant status, parental marital status, and family economic status, which could offer a general picture of how self-harming and suicidal behaviors may vary across different groups. 

Secondly, to investigate whether gender, age, immigration status, and positive youth development are predictive of adolescent self-harm and suicidal behaviors, two logistic regression models were tested using the probability of having self-harm behaviors and the probability of attempting suicide as the outcome variables, respectively. Specifically, basic demographic factors (age and gender) were entered in the first block, family economic status, parental marital status, and immigration status of the participant were entered into the second block, general family functioning was entered in the third block; and finally the participant's academic and school competence (ASC) and positive youth development (CPYDS) were entered into the fourth block. In the first model, whether the participants had displayed any of the listed self-harm behaviors served as the dependent variable. For the second model, the probability of showing any suicidal behavior (i.e., suicidal thinking, suicidal plan, or suicidal attempt) was the dependent variable. 

Because of the high correlations between CPYDS and different second-order positive youth development factors, two additional logistic regression analyses were conducted with the four second-order factors of positive youth development, including cognitive behavioral competence (CBC), prosocial attributes (PA), positive identity (PIT), and general positive youth development qualities (GPYDQ), being entered into the fourth block of the above regression models. The purpose was to examine the respective effects of different positive youth development constructs to adolescent self-harming and suicidal behaviors. 

It should be noted that while examining the relationship between immigration status and self-harm or suicidal behaviors, immigrant youth who were born in other places than mainland China were not included in the analyses because they only accounted for 1.9% of the participants. In other words, the present study focused on comparing local and immigrant adolescents from mainland China. In addition, as 465 participants did not indicate whether their family was receiving CSSA in the year of survey or not, they were not included in the regression analyses.

## 3. Results

### 3.1. Prevalence of Self-Harm and Suicidal Behavior in Hong Kong Adolescents

Numbers and percentages of participants who reported having different self-harm behaviors in the past one year are presented in Table 2, which provides a general picture of self-harm behaviors in Hong Kong adolescents. Several observations can be highlighted from the findings. First, self-harm behavior was common in Hong Kong adolescents. Among the participated students, 1,087 respondents (32.7%) endorsed at least one form of deliberate self-harm behaviors in the past one year. Second, the most prevalent self-injurious behavior was “severely scratch oneself to the extent that scarring or bleeding occurred,” reported by 10.4% of the participants. Other popular forms of self-injury included “intentionally prevent wounds from healing” (8.6%), “cut one's wrist, arms, or other areas of one's body” (8.3%), and “bite oneself to the extent that one's skin is broken” (7.4%). Third, severe self-harm behaviors, such as “break one's own bones” (0.6%), “drip acid onto one's skin” (0.3%), and “use bleach, comet, or oven cleaner to scrub one's skin” (0.5%), were relatively uncommon. 

 Participants' suicidal behaviors in terms of suicide thought, suicide plan, and suicide attempt are summarized in Table 3. Specifically, 13.7% of the adolescents (446) reported that they had seriously thought about attempting suicide; 4.9% (158) had made specific suicidal plans, and 4.7% (152) had actually attempted suicide during the past year. Of the 152 students who attempted suicide, approximately 15% reported that their attempts had resulted in an injury or poisoning that required medical treatment. 

### 3.2. Psychosocial Correlates of Self-Harm and Suicidal Behaviors

Means and standard deviations of DSH and SB in different groups of gender, immigrant status, parental marital status, and family economic status are first summarized in Table 4. Two sample *t*-tests and one-way ANOVA were conducted to compare students' scores on DSH and SB among different groups. Several observations can be highlighted from the results. First, female students scored significantly higher than did male students in both DSH and SB, suggesting that girls were more likely to display self-harming and suicidal behaviors than were boys. Second, adolescents whose family receiving CSSA had relatively higher scores on SB than did students whose family did not receive CSSA, which indicates a possible role of low family economic status in adolescent suicidal behaviors. Third, local youth and immigrant adolescents did not differ in their DSH and SB scores. Fourth, parental marital status significant predicted both DSH and SB. Post hoc analyses suggested that adolescents whose parents were divorced and remarried with other people scored significantly higher than did adolescents whose parent were married couple. These results provide a rough picture of how different demographic factors may affect adolescent self-harming and suicidal behaviors.

Table 5 presents the simple correlations among family functioning, positive youth development constructs, and deliberate self-harm. As predicted, general family functioning was negatively correlated with self-harming and suicidal behaviors while positively correlated with all positive youth developmental indicators. Both the general indicator and different second-order factors of positive youth development were negatively correlated with self-harming behavior and suicide. These relationships were basically consistent with the literature and the hypotheses. 

The results of logistic regression analyses on self-harm behaviors (DSH) are shown in Table 6. There are several significant findings. First, gender predicted the probability of displaying self-destructive behaviors. The occurrence of self-injury was about 1.3-times higher among female students in comparison with male students (OR = 1.32, *P* =  .01). Second, students who reported higher level of family functioning were less likely to show self-harm behaviors as compared to students whose family functioning was low (OR = 0.73, *P* <  .001). Third, there was a negative relationship between participants' overall positive youth development and the occurrence of self-harm behaviors; the higher the positive youth development, the lower the risk of self-harm behaviors (OR = 0.72, *P* <  .001). Fourth, higher academic and school competence was associated with lower occurrence of self-harm behaviors (OR = 0.79, *P* <  .001). 

To determine whether different aspects of positive youth development would contribute to adolescent self-harming behaviors differently, a separate regression model was tested with four second-order factors of positive youth development instead of CPYDS being entered into the fourth block of the initial model. As shown in Table 6, higher academic and school performance still predicted lower rates of self-harming behavior in this model (OR = 0.80, *P* =.01); general positive youth development qualities (GPYDQ) were negatively correlated with the occurrence of self-harm behaviors (OR = 0.55, *P* <  .001). Unexpectedly, CBC positively contributed to DSH (OR = 1.26, *P* =  .04), suggesting that there was an increase in the risk of self-injury in adolescents with higher cognitive behavioral competence.

Table 7 presents the results for the prediction of suicidal behavior (SB). First, adolescent girls were two-times more likely to engage in suicidal behavior than did adolescent boys (OR = 2.07, *P* <  .001). Second, the risk of suicide in adolescents whose parents were remarried with other persons was 2.53-times (*P* <  .001) higher than adolescents from intact families. Third, adolescents with higher academic and school competence (OR = 0.76, *P* =  .01) demonstrated lower rate of risk for suicidal behavior. Fourth, higher overall positive youth development was associated with lower rate of suicidal behavior (OR = 0.55, *P* <  .001). It should be noted that the effect of family economic status on suicidal behavior was nonsignificant in the regression model after other demographic variables being controlled. This suggests that the higher score of suicidal behavior in adolescents with lower family economic status found in the *t*-test results may be due to the moderating effects of other variables such as gender and parental marital status. 

Results of the additional analyses also showed that higher levels of academic and school performance and general positive youth development qualities significantly predicted lower levels of suicidal behavior. In particular, the risk of suicidal behavior would decrease by approximately three-times when participants' scores on general positive youth development increase one point (OR = 0.24, *P* <  .001). This suggests that general positive youth development is a strong protective factor for adolescent suicide. Again, contrary to our expectation, higher cognitive and behavior competence was related to higher occurrence of suicidal behavior. The odds ratio was 1.94 (*P* <  .001), which means that one point increased in the participant's cognitive behavioral competence score would almost double the risk of suicidal behavior for the participants.

## 4. Discussion

The present study explored the prevalence and psychosocial correlates of deliberate self-harm (DSH) and suicidal behavior among Chinese youth based on a large sample of Hong Kong secondary school students. The results suggested that both DSH and suicidal behavior were not rare among young people in Hong Kong. Several risk and protective factors were identified. First, being female and having remarried parents increased the likelihood of displaying DSH and suicidal behavior. Second, higher level of family functioning decreased the incidence of DSH and suicidal behavior. Third, academic and school competence and overall positive youth development were negatively related to the occurrences of both self-harming and suicidal behavior. These findings are basically consistent with our hypotheses and provide important information for developing evidence-based prevention strategies for DSH and suicidal behavior in Chinese adolescents.

In this study, 32.7% of the participants reported at least one form of DSH within the previous year. The prevalence rate was comparable to the figures reported in the United States [6, 7], but it was apparently higher than in previous studies conducted among adolescents in different Chinese communities [47, 48]. This discrepancy might be an indicator for the increased incidence of DSH in Chinese youth. Another possible explanation may be that in contrast to previous studies, a more comprehensive measure of DSH that includes as many as 17 forms of self-harming behaviors was used in the present study, which perhaps increased the opportunity that various self-harming behaviors being disclosed. The prevalence of suicidal behaviors was found to be basically congruent with prior report. Around 13.7% of the adolescents reported having suicidal thoughts and 4.9% had suicidal plans in the past 12 months. It should be noted that among adolescents with suicidal plans (4.9%), almost all of them (4.7%) attempted suicide and 15% of the attempts were serious to the extent that medical treatment was required. These figures provide important information for helping professionals and policy makers in Hong Kong and other Chinese societies that DSH and suicidal behaviors are becoming an increasingly serious health problem for adolescents and thus timely intervention/prevention strategies are sorely needed. 

Consistent with previous findings, adolescent girls were found to have significantly higher rates of DSH and suicidal behaviors than adolescent boys. Specifically, the occurrence of DSH was about 1.3-times higher among female students in comparison with male students; and girls were about 2.1-times more likely to show suicidal tendency. Most researchers have explained gender difference in DSH and suicidal behaviors in terms of the role of gendered socialization experiences [49]. For example, Clery argued that males learn to direct conflicts externally while women learn to turn their anger inward. As such, male aggression tends to be criminalized and female aggression tends to be expressed in the forms of internalized problems, such as self-injury [50]. To further understand what factors may account for gender difference in DSH and suicide-related behavior in adolescents, the meaning of the two types of behaviors in the lives of male and female youth should be further investigated. It should be noted that although successful suicide has been found to occur more frequently in males than in females [51], the present study only focused on the less extreme forms of suicidal behaviors (unsuccessful suicide), that is, suicidal thoughts, plans, and attempts. Further research is needed to examine gender difference in real suicide in Chinese adolescents. 

Two family factors were found to have predictive effects on adolescent DSH and suicidal behavior: low level of family functioning (i.e., low family mutuality, low family communication and high family conflict) and having remarried parents increased the likelihood of these two kinds of risky behaviors. While there is abundant evidence supporting the protective role of good communication and harmonious relationships among family members for adolescent development [34], family conflict and parental marital discord have long turned out to be salient risk factors for emotional and behavioral problems in youth, because family conflict creates particularly stressful life experiences for children and adolescents [52, 53]. As a way of coping, adolescents use self-harm to release intense, overwhelming negative emotions and obtain a brief escape from the distress, to get a sense of control over the pain and their lives, and sometimes to deal with strong feelings of guilt, shame, or self-hatred [54]. Desperate avoidance such as suicide and anger-based delinquency may also occur under such circumstances [55, 56]. In line with prior findings, the present results provide further support for the important role of family experiences in Chinese adolescents' self-harming and suicidal behaviors. Therefore, it seems necessary to involve units that target at improving various family factors in designing relevant prevention/intervention programs, such as building up supporting family relationships, reducing family conflicts, and providing support for different family members in nonintact family, especially children.

As expected, positive youth development significantly predicted DSH and suicidal behavior among Chinese adolescents. Higher levels of overall positive youth development and academic performance were closely related to lower rates of DSH and suicidal behaviors. The effects of positive youth development in adolescent promotion/prevention programs have been investigated by many researchers. In the United States, Catalano et al. identified 25 effective programs focusing on positive youth development [44]. These programs all demonstrated robust and sustained impacts on promoting positive outcomes and preventing problem behaviors (e.g., substance abuse, delinquency, campus violence, and youth pregnancy) among adolescents. In Hong Kong, Shek and colleagues [57] developed a multiple-year positive youth development program for Chinese adolescents, the Project P.A.T.H.S. After six years of implementation of the program in more than 200 secondary schools in Hong Kong, significant program effects were shown in multiple areas, including reduced adolescent problem behaviors (e.g., Internet addiction, delinquent behaviors, consumption of pornographic readings, and substance abuse) and increased positive developmental outcomes (e.g., bonding with others, emotional and social competence, self-efficacy, and prosocial norms) [39, 41, 58]. Aligning with these findings, the present finding that positive youth development was negatively related to the likelihood of showing self-harming or suicidal behaviors provides direct support for the utilization of positive youth development programs in preventing adolescent risk behaviors. 

Several unexpected findings were also observed. First, family economic status was related to neither adolescent DSH nor suicidal behavior. Second, immigrant students had no higher rates of DSH and suicidal behaviors than did local students. These results seemed to be inconsistent with prior findings that that low family economic status and immigrant status often increased the vulnerability of self-harm and suicide in adolescents [59] due to the heightened exposure to multiple stressors [60], lack of material and social resources and support [61], and social exclusion caused by lack of family assets and/or immigrant identity [62]. One possible reason may be that family economic status was indexed by one item (i.e., whether the student was from a family with CSSA), so that the real economic condition of the family may not be fully reflected in the response. In future studies, multiple items and more quantitative measures of family economic status could be used. For immigrant status, it is possible that there are other variables moderating or mediating its effects on youth DSH and suicidal behaviors. Researchers have found that immigrant youth from China mainland to Hong Kong had higher levels of self-control, empathy, assertiveness and ability to read social cues which led to their better mental health than local youth [63]. To investigate which factors and how they affect the adaption of immigrant youth and prevent them from various risk behaviors could be a direction for future research. 

Third, one positive youth development construct, cognitive and behavioral competence, was positively related to the occurrence of DSH and suicidal behavior. This finding is contradictory to the common finding that positive youth development helps to reduce problem behaviors. In this study, the measured cognitive and behavioral competence involves three aspects: cognitive competence, behavioral competence, and a sense of self-determination. According to Catalano et al. [44], while high levels of cognitive competence and behavioral competence enable one to develop skills for self-understanding, problem-solving, and making effective behavioral choices, a high sense of self-determination allows an individual to think for oneself and to take action consistent with that thought. In this sense, if people believe that self-harm or suicide could be an effective way to cope with painful events and to relieve one's sufferings, those with higher self-determination may be more likely to take real action, that is, commit self-harming or suicidal behaviors. This possibility can be further explored by separating self-determination from the general cognitive and behavioral competence and examining their respective relationships with different problem behaviors. On the other hand, the present finding also suggests that, to effectively prevent youth problem behaviors, different aspects/constructs of positive youth development must be developed concurrently. For example, when we try to foster a sense of self-determination in adolescents, we must also teach them skills that help to manage one's emotion (emotional competence), to adapt to change and stressful events in healthy and flexible ways (resilience), and to develop commitment to social relationships in the family, school, and culture (bonding). The interactive effects among different positive youth development constructs on preventing adolescent risk behaviors could be tested in future research. 

This study examined the prevalence of deliberate self-harm (DSH) and suicidal behaviors among Chinese adolescents based on empirical research. The high prevalence of DSH and suicidal behavior in Hong Kong secondary school students could be considered a forewarning to researchers, school practitioners, and public health professionals in Hong Kong society, and perhaps in other Chinese communities. Several correlates of the two phenomena were identified, including gender, family functioning, parental marital status, academic and school competence, and positive youth development. Such knowledge is useful to help researchers and professionals better understand, predict, prevent and treat DSH and suicidal behaviors among Chinese adolescents. In addition, the fact that DSH and suicidal behavior shared similar associated factors provides further support for the preventive approach that addresses both risk and protective factors for multiple problems simultaneously, such as the Project P.A.T.H.S.

## Figures and Tables

**Table 1:** Descriptive statistics about participants.

Categorical variables	*n*	%		
Gender				
Male	1,719	52.2%		
Female	1,572	47.8%		
Place of birth				
Hong Kong	2,590	78.3%		
Mainland China	655	19.8%		
Others	64	1.9%		
Parental marital status			
First marriage	2781	84.4%	
Divorced	209	6.3%	
Separated	73	2.2%	
Remarried	129	3.9%	
Others (not first marriage)	104	3.2%	
Family economic status				
Receiving CSSA	225	6.8%		
Not receiving CSSA	2,606	78.3%		
Others	465	13.9%		

Continuous variables	Mean	SD	Range	Cronbach's *α*

Age	12.59	0.74	10–18	—
CFAIALL	3.73	0.81	1–5	0.90
CPYDS	4.51	0.70	1–6	0.96
ASC	3.12	0.67	1–5	0.67
CBC	4.45	0.75	1–6	0.87
PA	4.50	0.89	1–6	0.83
GPYDQ	4.50	0.71	1–6	0.93
PIT	4.24	0.96	1–6	0.87
DSH	0.04	0.10	0-1	0.83
SB	0.08	0.21	0-1	0.68

CSSA = comprehensive social security assistance; CFAIALL = general family interaction; CPYDS = positive youth development; ASC = academic school competence; CBC = cognitive behavioral competence; PA = prosocial attributes; GPYDQ = general positive youth development qualities; PIT = positive and clear identity; DSH = deliberate self-harm; SB = suicidal behavior.

**Table 2:** Percentage of participants with self-harming behavior.

During the past year, did you ever intentionally any of the following	No		Yes	
	Number	Percent	Number	Percent
(1) Cut your wrist, arms, or other area(s) of your body?	3042	91.7%	277	8.3%
(2) Burned yourself with a cigarette?	3297	99.2%	26	0.8%
(3) Burned yourself with a lighter or a match?	3274	98.7%	42	1.3%
(4) Carved words into your skin?	3153	95.1%	161	4.9%
(5) Carved pictures, designs, or other marks into your skin?	3135	94.9%	169	5.1%
(6) Severely scratched yourself, to the extent that scarring or bleeding occurred?	2956	89.6%	343	10.4%
(7) Bit yourself, to the extent that you broke the skin?	3045	92.6%	244	7.4%
(8) Rubbed sandpaper on your body?	3263	99.1%	30	0.9%
(9) Dripped acid onto your skin?	3279	99.7%	10	0.3%
(10) Used bleach, comet, or oven cleaner to scrub your skin?	3259	99.5%	16	0.5%
(11) Stuck sharp objects such as needles, pins, and staples, into your skin (not including tattoos, ear piercing, needles used for drug use, or body piercing)?	3158	96.8%	103	3.2%
(12) Rubbed glass into your skin?	3178	98.4%	52	1.6%
(13) Broken your own bones?	3203	99.4%	20	0.6%
(14) Banged your head against something, to the extent that you caused a bruise to appear?	3060	95.7%	138	4.3%
(15) Punched yourself, to the extent that you caused a bruise to appear?	2992	94.2%	184	5.8%
(16) Prevented wounds from healing?	2876	91.4%	272	8.6%
(17) Done anything else to hurt yourself that was not asked about in this questionnaire?	2986	95.3%	148	4.7%

Participants who had shown any of the above self-harm behaviors	**2241**	**67.3%**	**1087**	**32.7%**

**Table 3:** Percentage of participants with suicidal behavior in the past year.

During the past one year	No		Yes	
	Number	Percent	Number	Percent
(1) Have you seriously thought about attempting suicide?	2816	86.3%	446	13.7%
(2) Did you make a specific plan about how you would attempt suicide?	3095	95.1%	158	4.9%
(3) Did you actually make a suicide attempt?	3095	95.3%	152	4.7*% *
(4) If you attempted suicide, did that attempt result in an injury or poisoning that had to be treated by a doctor or nurse?	3206	99.3%	22	0.7%

Participants who had shown any of the above suicidal behaviors	**2758**	**85.3%**	**476**	**14.7%**

**Table 4:** Means and standard deviations of self-harm and suicidal behavior in different groups by gender, immigrant status, family economic status, and parental marital status.

	DSH				SB			
	Mean	SD	Statistics	*P*	Mean	SD	Statistics	*P*
Gender								
Male	.03	.10	*t* = −2.23	**.03**	.05	.14	*t* = −5.40	**.00**
Female	.04	.10			.08	.18		
Immigrant status								
Hong Kong	.04	.10	*t* = −1.10	.27	.07	.16	*t* = 1.56	**.12**
Mainland China	.03	.09			.06	.17		
Family economic status								
Not receiving CSSA	.04	.09	*t* = 0.34	.74	.06	.15	*t* = 2.34	**.02**
Receiving CSSA	.04	.10			.09	.19		
Parental marital status								
Divorced	.05	.13	*F* = 4.48	**.00** ^**a**^	.07	.17	*F* = 7.94	**.00** ^**b**^
Separated	.05	.09			.09	.19		
Married	.04	.09			.05	.15		
Remarried	.07	.13			.12	.22		
Others	.05	.12			.09	.19		

^a^Tukey's HSD post hoc test showed that significant difference in DSH exists between married and remarried parental marital status.

^b^Tukey's HSD post hoc test showed that significant difference in SB exists between married and remarried parental marital status.

**Table 5:** Correlations among continuous variables.

	CFAIALL	CPDYS	ASC	CBC	PA	GPYDQ	PIT	DSH
CFAIALL	—	—	—	—	—	—	—	—
CPDYS	.53	—	—	—	—	—	—	—
ASC	.28	.38	—	—	—	—	—	—
CBC	.37	.88	.26	—	—	—	—	—
PA	.42	.81	.28	.60	—	—	—	—
GPYDQ	.55	.97	.37	.79	.74	—	—	—
PIT	.42	.84	.40	.71	.63	.75	—	—
DSH	−.24	−.25	−.14	−.18	−.19	−.25	−.20	—
SB	−.26	−.24	−.13	−.14	−.19	−.26	−.18	.43

CFAIALL = general family interaction; CPYDS = positive youth development; ASC = academic school competence; CBC = cognitive behavioral competence; PA = prosocial attributes; GPYDQ = general positive youth development qualities; PIT = positive and clear identity; DSH = deliberate self-harm; SB = suicidal behavior.

All correlations are significant at the 0.01 level.

**Table 6:** Logistic regression analyses on participants' self-harm behavior.

	B	Odds ratio	*P*
First block			
Age	0.07	1.07	.36
Gender	0.28	1.32	.01
Second block			
Immigration status	−0.13	0.88	.33
Family economic status (CSSA)	−0.18	0.83	.37
Parental marital status			
Parent_divorced	0.18	1.19	.41
Parent_separated	0.09	1.10	.79
Parent_remarried	0.18	1.20	.47
Parent_others	0.30	1.35	.29
Third block			
CFAALL	−0.31	0.73	.00
Fourth block			
ASC	−0.23	0.79	.00
CPYDS	−0.33	0.72	.00

Additional analyses^a^	B	Odds ratio	*P*

Fourth block			
ASC	−0.22	0.80	.01
CBC	0.23	1.26	.04
PA	−0.01	0.99	.90
GPYDQ	−0.60	0.55	.00
PIT	0.00	1.00	.97

^a^In additional analyses, four second-order factors of positive youth development (CBC, PA, GPYDQ, and PIT) were used to replace CPYDS in the fourth block of the regression model. Other variables in the regression model and the order of entry were the same as the initial model.

Immigration status: 1 = immigrant from mainland China; 0 = local participant

Family economic status: 1 = receiving comprehensive social security assistance (CSSA); 2 = not receiving CSSA

Parent_divorced: 1 = divorced; 0 = married (first marriage)

Parent_separated: 1 = separated; 0 = married (first marriage)

Parent_remarried: 1 = remarried; 0 = married (first marriage)

Parent_others: 1 = others (not first marriage); 0 = married (first marriage)

CFAIALL = general family interaction; ASC = academic school competence; CPYDS = positive youth development; CBC = cognitive behavioral competence; PA = prosocial attributes; GPYDQ = general positive youth development; PIT = positive and clear identity.

**Table 7:** Logistic regression analyses on participants' suicidal behavior.

	B	Odds ratio	*P*
First block			
Age	−0.01	0.99	.95
Gender	0.73	2.07	.00
Second block			
Immigration status	−0.01	0.99	.95
Family economic status (CSSA)	0.34	1.41	.16
Parental marital status			
Parent_divorced	−0.09	0.92	.76
Parent_separated	−0.59	0.55	.24
Parent_remarried	0.93	2.53	.00
Parent_others	0.02	1.02	.95
Third block			
CFAIALL	−0.53	0.59	.00
Fourth block			
ASC	−0.27	0.76	.01
CPYDS	−0.60	0.55	.00

Additional analyses ^a^	B	Odds ratio	*P*

Fourth block			
ASC	−0.26	0.77	.02
CBC	0.66	1.94	.00
PA	−0.05	0.95	.67
GPYDQ	−1.42	0.24	.00
PIT	0.09	1.09	.42

^a^In additional analyses, four second-order factors of positive youth development (CBC, PA, GPYDQ, and PIT) were used to replace CPYDS in the fourth block of the regression model. Other variables in the regression model and the order of entry were the same as the initial model

Immigration status: 1 = immigrant from mainland China; 0 = local participant Enter Family economic status: 1 = receiving comprehensive social security assistance (CSSA); 2 = not receiving CSSA

Parent_divorced: 1 = divorced; 0 = married (first marriage)

Parent_separated: 1 = separated; 0 = married (first marriage)

Parent_remarried: 1 = remarried; 0 = married (first marriage)

Parent_others: 1 = others (not first marriage); 0 = married (first marriage)

CFAIALL = general family interaction; ASC = academic school competence; CPYDS = positive youth development;

CBC = cognitive behavioral competence; PA = prosocial attributes; GPYDQ = general positive youth development; PIT = positive and clear identity.
